# MyBP-C: one protein to govern them all

**DOI:** 10.1007/s10974-019-09567-1

**Published:** 2020-01-20

**Authors:** L. W. H. J. Heling, M. A. Geeves, N. M. Kad

**Affiliations:** School of Biosciences, University of Kent, Canterbury, CT2 7NH UK

**Keywords:** cMyBP-C, Muscle contraction, Contractility, Cardiac, Myosin binding protein C

## Abstract

The heart is an extraordinarily versatile pump, finely tuned to respond to a multitude of demands. Given the heart pumps without rest for decades its efficiency is particularly relevant. Although many proteins in the heart are essential for viability, the non-essential components can attract numerous mutations which can cause disease, possibly through alterations in pumping efficiency. Of these, myosin binding protein C is strongly over-represented with ~ 40% of all known mutations in hypertrophic cardiomyopathy. Therefore, a complete understanding of its molecular function in the cardiac sarcomere is warranted. In this review, we revisit contemporary and classical literature to clarify both the current standing of this fast-moving field and frame future unresolved questions. To date, much effort has been directed at understanding MyBP-C function on either thick or thin filaments. Here we aim to focus questions on how MyBP-C functions at a molecular level in the context of both the thick and thin filaments together. A concept that emerges is MyBP-C acts to govern interactions on two levels; controlling myosin access to the thin filament by sequestration on the thick filament, and controlling the activation state and access of myosin to its binding sites on the thin filament. Such affects are achieved through directed interactions mediated by phosphorylation (of MyBP-C and other sarcomeric components) and calcium.

## Introduction

Muscle contraction and relaxation on the molecular level is achieved by the sliding movement of interdigitating thick filaments containing myosin and thin filaments containing actin in the sarcomere. Fundamentally, this process is driven by the cyclic interaction between myosin heads and actin filaments coupled with ATP hydrolysis and conformational changes of the myosin head (Geeves and Holmes 1999). Aside from actin and myosin the sarcomere contains an array of additional proteins that aid in the assembly or integrity of the sarcomere, and regulate the force, rate and timing of contraction.

## Structure/localisation

**Fig. 1:**
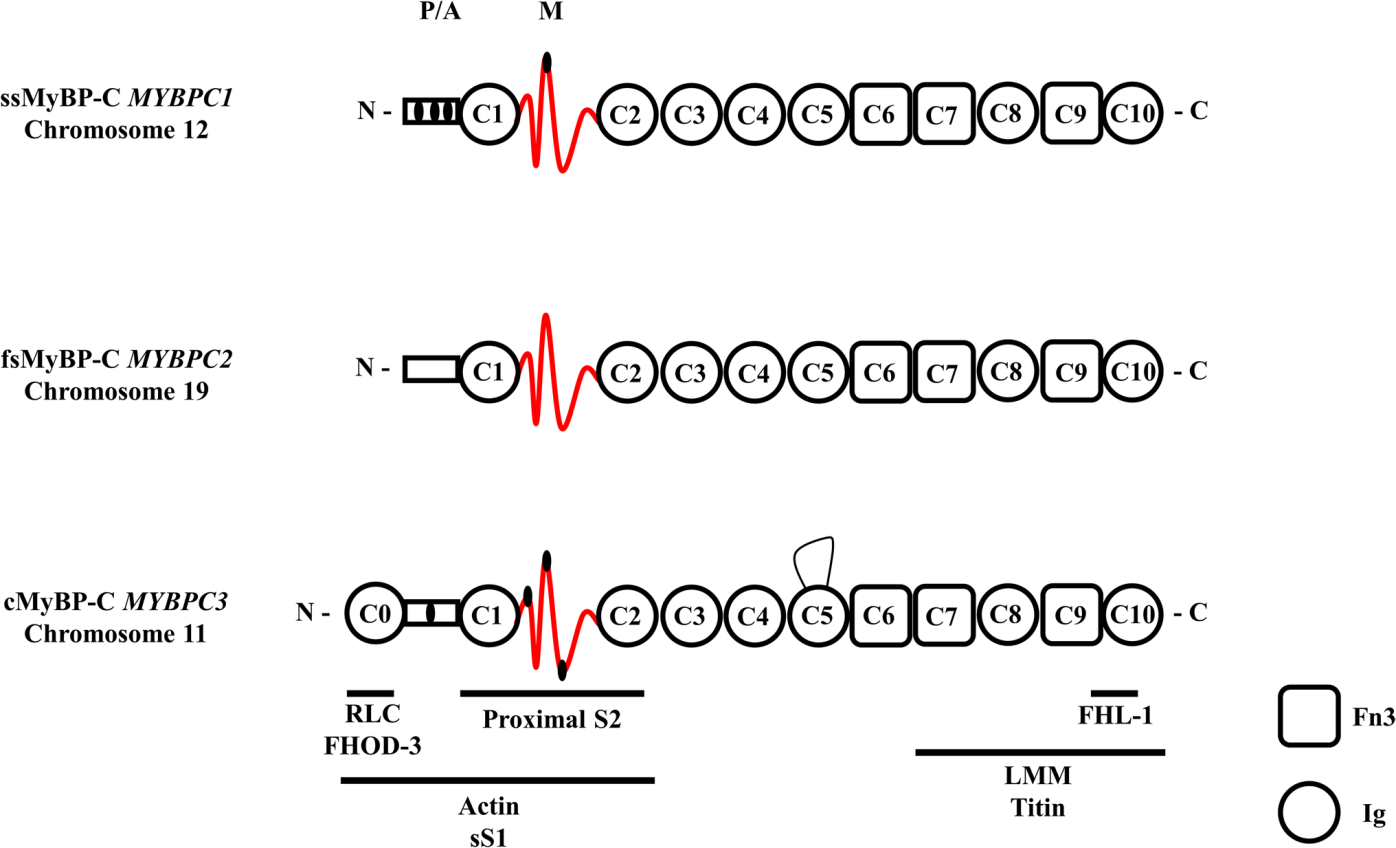
Schematic diagram of full length slow skeletal (ss), fast skeletal (fs) and cardiac (c) MyBPC paralogs. Each isoform comprises three Fn3 domains and seven or eight Ig domains. The known binding partners and positions are indicated by the horizontal stripes below. Note the phosphorylation sites in the P/A and M domain of the ssMyBP-C and cMyBP-C paralogs are indicated by small black ellipses. The cMyBP-C has an additional 28 amino acid loop in the C5 domain

Myosin binding protein C (MyBP-C) is a sarcomeric accessory protein that was first identified as a contaminant of crude skeletal muscle preparation (Starr and Offer 1971). The protein is approximately 40 nm in length, 3 nm in width, and has a molecular weight of ~ 140 kDa (Hartzell and Sale 1985). There are three paralogs, encoded by three different genes on different chromosomes (Fig. 1). Slow skeletal (ss)MyBP-C is encoded by *MYBPC1* on chromosome 12, fast skeletal (fs)MyBP-C by *MYBPC2* on chromosome 19 and cardiac (c)MyBP-C by *MYBPC3* on chromosome 11. cMyBP-C was discovered after ssMyBP-C and fsMyBP-C (Hartzell and Titus 1982; Yamamoto and Moos 1983), and as the name suggests is exclusive to cardiac muscle.

The three paralogs have likely arisen through gene duplication and have similar primary structures; cardiac shares 54.4% sequence identity with fast skeletal, and for slow skeletal the identity to cardiac is 52.4% (Weber et al. 1993; Yasuda et al. 1995). The proteins are primarily formed of a series of globular domains of the immunoglobulin (Ig) or fibronectin-III (Fn3) families named C1–C10 from the N-terminus with an additional motif or M-domain linking C1 and C2 and a proline alanine rich region (P/A) at the N-terminus (Fig. 1). cMyBP-C has an additional Ig domain C0 at the N-terminus and has 4 serine residues that can be phosphorylated in the M-domain (Yasuda et al. 1995) as well as an additional 28 amino acid loop in the C5 domain (Flashman et al. 2004).

Two studies in 1995 linked mutations on *MYBPC3* to familial hypertrophic cardiomyopathy (HCM) (Bonne et al. 1995; Watkins et al. 1995), a disease that affects 1 in 200 people and the most common cause of sudden death in young people (Harvey and Leinwand 2011; Maron and Maron 2013; Semsarian et al. 2015). Currently, 40% of the sarcomeric mutations known to be linked to HCM have been found in cMyBP-C (Carrier et al. 2015). The gradual emergence of the link between cMyBP-C and HCM has shifted focus towards understanding the structure and function of cMyBP-C in disease and normal physiology. This protein is not essential for viability, confirmed in mouse knockout studies, however, significant deficits in contraction were observed, indicating a modulatory role in contraction (Harris et al. 2002). We outline some of the ways this could be achieved below. MyBP-C’s other potential roles range from physiological to structural. Several reports have described MyBP-C binding partners in the sarcomere that may offer a contribution to their formation, maintenance and general function. Calmodulin has been reported to interact with cMyBP-C. This interaction may regulate the binding and unbinding of cMyBP-C to myosin proximal subfragment-2 by initiating rapid phosphorylation of Ca^2+^/calmodulin dependent kinase II (CaMKII) targets on MyBP-C or inducing phosphorylation of the RLC by myosin light chain kinase (Lu et al. 2012). The presence of a Ca^2+^/calmodulin binding site in the N-terminal region of cMyBP-C raises questions about the role of this Ca^2+^-signalling pathway in each contraction cycle. Does Ca^2+^ induce a response in cMyBP-C beat to beat, or is there a longer-term integration of the calcium signal by down-stream phosphorylation events?

Another binding partner to MyBP-C is Four and a Half LIM protein 1 (FHL1 or SLIM1), a highly expressed protein in skeletal and cardiac muscle. MyBP-C seems integral for the incorporation of FHL1 into the thick filament (McGrath et al. 2006). While the exact function of FHL1 is unknown, overexpression and knockout results in poor sarcomere assembly. Mutations in muscle LIM protein have been linked to dilated cardiomyopathy (DCM) (Knoll et al. 2002). Mutations in MyBP-C can therefore have effects downstream through its interactions with these auxiliary proteins.

More recently cardiac formin Fhod3 has been reported to have strong binding interactions (K_d_: 0.8 μM) with the cMyBP-C (Matsuyama et al. 2018). Like MyBP-C, Fhod3 is localised to the C-zones of the A-band, and MyBP-C is necessary for this localisation. Fhod3 contributes to actin polymerization, nucleation and recruitment of profilin actin dimers (Blanchoin et al. 2014). It also has an important role in regulating actin assembly in the sarcomere and maintaining cardiac function in perinatal and adult hearts (Ushijima et al. 2018). This further supports the notion that cMyBP-C has multiple roles ranging from structural organisation to the Ca^2+^ response, and explains why mutations in MyBP-C can have downstream effects in general sarcomere maintenance. For the physiological and structural role of MyBP-C in sarcomere maintenance we direct readers to more specialized reviews of (Flashman et al. 2004; Harris et al. 2011; Moss et al. 2015).

Here we will discuss the current knowledge in the field about MyBP-C and its interactions with other sarcomere proteins. In particular, we will focus on the interplay between MyBP-C and myosin, MyBP-C and actin and consider the potential complexities that this brings. The inevitable ebb and flow of research activity has led to interest swinging between the MyBP-C interaction with the thick or thin filament. We emphasise here why it is important to bring these together to provide an informed opinion on how this important cardiac regulator functions.

## C-terminal interactions of cMyBP-C with myosin

**Fig. 2:**
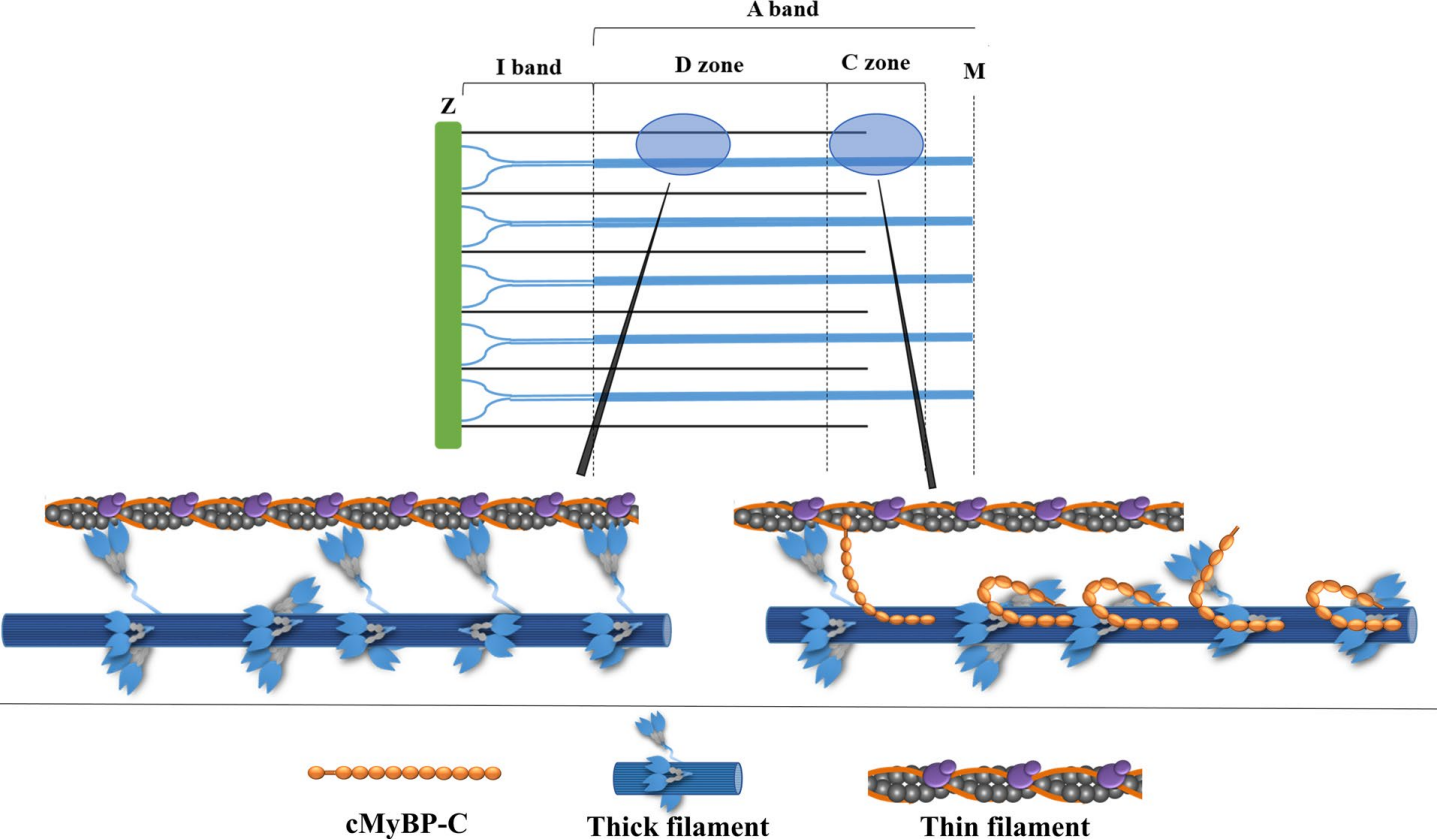
Schematic diagram of half-sarcomere organisation. The A-band contains thick filaments and overlapping thin filaments, the C-zone is the region of the thick filament with MyBP-C present, and the D-zone has no MyBP-C. The I-band contains thin filaments and also titin, which leads from the Z-line into the thick filaments. These areas are shown with more detail below, highlighting the content of the thick filaments in zone C versus D. For clarity only every third myosin crown is shown, please refer to Fig. 3 for details on stoichiometry

MyBP-C’s location in the sarcomere is limited to the C-zones of the A band (Fig. 2), regularly patterned in 7–9 transverse parallel stripes each containing 3 molecules and approximately 43 nm apart, which correspond with the axial repeat of myosins along the thick filament (Craig and Offer 1976; Luther et al. 2011). This results in a ratio of MyBP-C: myosin in the C-zone of 1:3. The two C-zones per thick filament cover a third to a half of the thick filament between the ends of the bare zone and the filament tips. This brings up the first unanswered questions about MyBP-C—what structural features confine MyBP-C to this section of the thick filament, and if location is linked to its regulatory role(s), why just this section?

The first identified binding partner of MyBP-C was myosin, as the name suggests. MyBP-C is anchored to the thick filament through strong binding interactions between the C7 and C10 domains and the light meromyosin (LMM) and titin backbone (Tonino et al. 2019). This interaction between MyBP-C and titin could explain the restriction of MyBP-C to the C-zone of the thick filament. C10 (of MyBP-C) binds to a short region of LMM (residues 1554-1581 (Human numbering)) through positively charged amino acids (Flashman et al. 2007), but C7–C9 are necessary to maximise binding affinity to between 0.5 and 3.5 µM depending on the isoform (Miyamoto et al. 1999; Okagaki et al. 1993) and key for localisation of MyBP-C to the A-band in the sarcomere (Gilbert et al. 1999). Different paralogs of MyBP-C have a higher affinity for the LMM and thick filament in the type of muscle where it is found, i.e. cardiac MyBP-C has higher affinity for cardiac LMM than for skeletal LMM (Alyonycheva et al. 1997).

The focus of the research on the binding properties for the N-terminal domains of MyBP-C have shifted several times over the decades. The C0–C7 domains are thought to extend from the thick filament and able to bind both the heavy meromyosin (HMM) region of the thick filament and the actin filament.

**Fig. 3:**
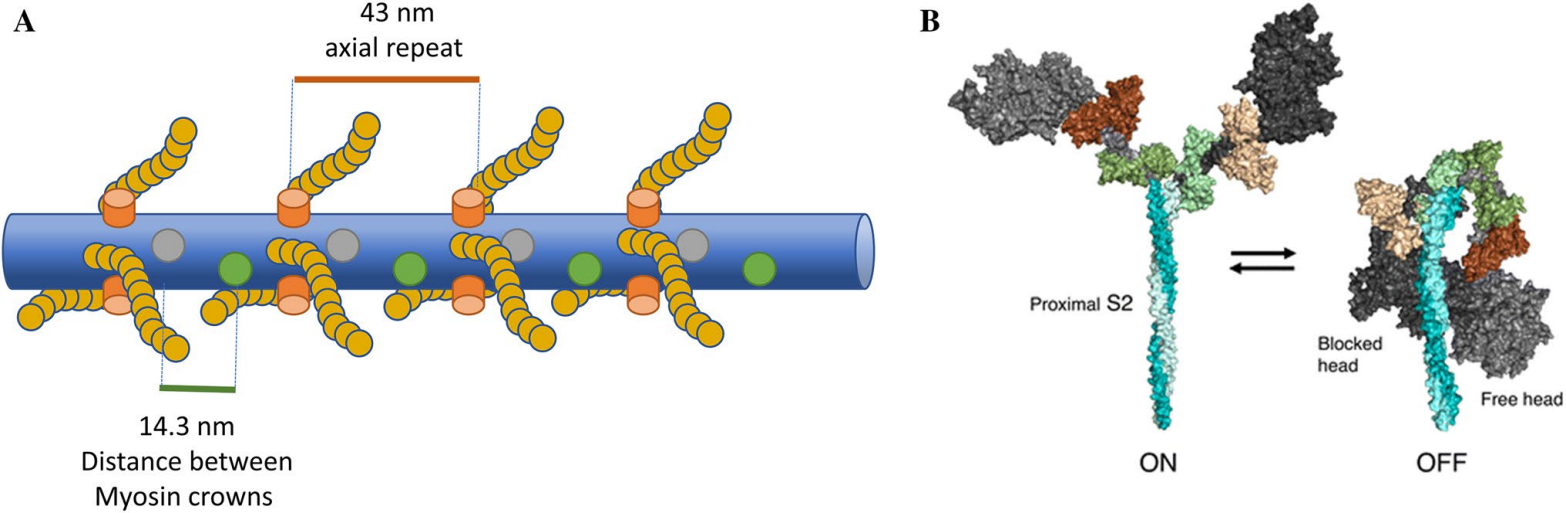
**a** The molecular arrangement of the thick filament in the C-zone. Three molecules of MyBP-C associate via their C-terminal regions with myosin S2 at every third crown (orange cylinders) leading to a 43 nm spacing between MyBP-C zones which matches the axial repeat. MyBP-C also interacts with myosin heads/neck through the N-terminal regions, although it is clear from the diagram that MyBP-C can possibly extend to other myosins in the 14.3 nm helical repeat therefore the precise myosin that interacts with MyBP-C is unknown. Discovering the hinge points of MyBP-C and the geometry of its arrangement on the thick filament will be integral for understanding how MyBP-C works. **b** Myosin can exist in two clear configurations, left (active. heads-unfolded), right (inactive, heads folded). MyBP-C can modulate these forms of myosin, although the mechanism and location of binding sites are not fully known. Image taken from (Trivedi et al. 2018)

An important aspect of the way in which MyBP-C can regulate the thick filament (and the thin filament) is the 3-D geometrical packing of the proteins into the thick filament. There are 3 MyBP-C for each 9 myosins in the C-zone. Thus, the arrangement is important for how MyBP-C may directly or indirectly interact with each pair of myosin heads (see Fig. 3). As stated above, the repeat pattern of myosins in the thick filament is 43 nm. Within each 43 nm are 9 myosins and 3 MyBP-C. The myosins are arranged in 3 sets of 3 crowns, 14.3 nm apart, with each crown rotated 40° around the thick filament (Zoghbi et al. 2008). The 43 nm repeat of the MyBP-C stripes therefore corresponds to three MyBP-C molecules at every third crown (Fig. 3a). For MyBP-C to control all myosins in the C-zone it must interact with the myosins in two additional crowns along the filament. MyBP-C affects the packing of myosin heads onto the backbone of the filament and that three crowns cooperate in both packing and activation such that one MyBP-C is sufficient to govern three myosins. EM images of the thick filament suggest possible interactions between the folded myosin heads and the axially adjacent pair of myosin heads away from the bare zone (Woodhead et al. 2005), but higher resolution structures of the thick filament structure are required to establish the detail of such contacts. Given the length of MyBP-C a strong interaction of the C0–C2 domains with a myosin head some distance from the binding site of the C10–C7 domains in the thick filament could sterically restrict additional myosins without the need for a specific binding site.

## MyBP-C phosphorylation

The functional effect of phosphorylation of the cMyBP-C M domain was first described in intact amphibian muscle (Hartzell 1984). Frog myocardium treated with isoproterenol to up-regulate phosphorylation showed an increase in tension which accelerated contraction and relaxation rates. The serine residues [Ser-273, Ser-282, Ser-302 and Ser-307; mouse sequence (Mohamed et al. 1998)] can be phosphorylated by an array of protein kinases, including protein kinase A (PKA) (Gautel et al. 1995; Mohamed et al. 1998), protein kinase C (PKC) (Mohamed et al. 1998), protein kinase D (PKD) (Bardswell et al. 2010), CaMKII (Gautel et al. 1995) and ribosomal S6 kinase (Cuello et al. 2011). cMyBP-C is highly phosphorylated under baseline conditions but phosphorylation is significantly reduced in many cardiac conditions like HCM (Sadayappan et al. 2005). This supports the physiological importance of cMyBP-C phosphorylation. During ischemia, dephosphorylated cMyBP-C was more prone to proteolysis than phosphorylated cMyBP-C (Sadayappan et al. 2005) and recently 29 kDa cMyBP-C fragments released during ischemia were shown to be useful early indicators of myocardial infarction (Govindan et al. 2013; Lyngbakken et al. 2019). It is difficult to distinguish the functional effects of phosphorylation of cMyBP-C from other adrenergic targets in the sarcomere, prioritising the use of transgenic models to further enhance our knowledge on these effects. One often used approach is to substitute the serine for aspartate residues to mimic phosphorylation. However it was shown using N-terminal MyBP-C fragments (C1-m-C2) that the functional and structural effects of phosphorylation in interaction with the thin filament are not mimicked by this substitution (Kampourakis et al. 2018). This is a point of consideration when analysing future results and this result will make the experimental study of phosphorylation effects significantly more challenging.

## Interactions between the N-terminus of cMyBP-C and myosin

The sarcomeric arrangement of myosin into thick filaments provides a large interaction area for cMyBP-C, which is mediated by the C-terminal domains as described above. However, the positioning of the remainder of the molecule is still under debate. Structural data indicates that MyBP-C stretches between the thick and thin filaments (Luther et al. 2011), and measurements of C0 binding to the regulatory light chain indicate an interaction of the very N terminus with myosin (Ratti et al. 2011). C1 has also been shown to interact with myosin adjacent to the light chains (Ababou et al. 2008), and C1–C2 binds S2 in a phosphorylation dependent manner (Gruen et al. 1999). These observations clearly suggest that the N-terminus of cMyBP-C modulates myosin, however it is not yet clear how and what is affected. Studies on the mouse myocardium revealed that loss of cMyBP-C resulted in an accelerated stretch activation response consistent with cMyBP-C suggesting an acceleration of the cross-bridge kinetics (Stelzer et al. 2006a, c), which was reversed by PKA phosphorylation (Stelzer et al. 2006c). Biochemical studies of isolated proteins suggest an enhancement of the actin activated ATPase at low MyBP-C concentrations that is offset by a greater reduction in ATPase at higher concentrations (Belknap et al. 2014). However, these effects could also be mediated by an interaction with actin, described in more detail below, rather than a direct effect of MyBP-C on myosin. To make the situation more complex, phosphorylation reduces the interaction between cMyBP-C and myosin with inotropic consequences (Gruen et al. 1999; Nag et al. 2017; Toepfer et al. 2013). Such observations would imply that unphosphorylated cMyBP-C inhibits myosin. Yet the basal level of MyBP-C phosphorylation is quite high (Gresham and Stelzer 2016). An interaction between cMyBP-C and a population of the super-relaxed state (SRX) of myosin likely explains this effect. However, the structure of this state still requires a clear definition. The SRX was originally defined as a thick filament state with suppressed ATPase (Hooijman et al. 2011), this does not have a defined structural correlate presently. However, conformations with myosin heads folded back onto one another (Fig. 3b) exist: the J-motif, originally identified in smooth muscle (Wendt et al. 2001) and the Interacting Heads Motif, seen in tarantula (Woodhead et al. 2005), scallop (Stafford et al. 2001) and limulus (Jung et al. 2008) and others (Lee et al. 2018) muscle, and correlate to turned off muscle. Such structures have also been seen in cardiac thick filaments (Zoghbi et al. 2008), suggesting that the SRX may correlate with these such structures. However, the SRX state has been found in HMM and myosin (Nag et al. 2017) suggesting that a state not packed onto the thick filament but still ‘off’ may exist (Caremani et al. 2019a). Nonetheless these ‘off’ heads reduce the population of force generating heads, decreasing the maximum tension that can be generated (McNamara et al. 2016; Spudich 2019; Starr and Offer 1978). As long as the number of heads still exceeds the duty cycle requirements then the maximum velocity will be affected to a lesser extent, if at all. Recently the binding site of MyBP-C has been mapped onto myosin in more detail and correlated with HCM mutations in *MYBPC3* to suggest an interaction interface across one face of myosin (the ‘mesa’) in the ordered relaxed conformation (Nag et al. 2017). Structural studies have shown that myosins leave the ‘off’ state to form the DRX (disordered relaxed state) state upon phosphorylation of the RLC (Colson et al. 2010; Levine et al. 1996) in striated muscle. These observations echo the effects of RLC phosphorylation on the regulation of tarantula muscle (Brito et al. 2011), and smooth muscle (Lowey and Trybus 2010; Wendt et al. 1999), which are exclusively thick filament regulated muscle types and possess no MyBP-C. However, in striated muscle, formation of the DRX is a compound effect of phosphorylation of the RLC (Levine et al. 1996; Stelzer et al. 2006b) and MyBP-C (Colson et al. 2010) as well as temperature and the fraction of the motor in the pre-powerstroke (M·ADP·Pi) conformation. The role and timing of each component is of considerable interest. RLC and MyBP-C phosphorylation appear to result in similar increases in the number of available heads, however lattice spacing appears to be affected more by RLC phosphorylation than effects of MyBP-C (Colson et al. 2007, 2010, 2012; Palmer et al. 2004) (although contested in Sadayappan et al. 2006), suggesting a distinct mechanism of force enhancement.

## N-terminal interactions of cMyBP-C with actin

MyBP-C’s interaction with actin was discovered shortly after its ability to bind myosin was determined (Moos et al. 1978). Since then, numerous experimental approaches have collectively shown the N-terminal region of MyBP-C is responsible for these interactions (Belknap et al. 2014; Harris et al. 2016; Inchingolo et al. 2019; Kensler et al. 2011; Lu et al. 2011; Luther et al. 2011; Mun et al. 2011, 2014; Orlova et al. 2011; Risi et al. 2018; Whitten et al. 2008). The N-terminal domains possess some interesting structural attributes; the Ig-like C0 domain is specific to the cardiac isoform and the proline-alanine rich region between C0 and C1 has sequences proposed to bind actin (Squire et al. 2003), that have been linked to modulating contractile velocity across species (Shaffer and Harris 2009). Also at the N-terminus is the M-domain, part of which has been shown to form a tri-helix bundle in isolation or with C2 (Howarth et al. 2012; Michie et al. 2016) and is structurally perturbed by Ca^2+^-calmodulin binding and phosphorylation (Michie et al. 2016; Previs et al. 2016). The precise regions of binding to actin are likely to vary between isoforms and species (Shaffer et al. 2010; van Dijk et al. 2014), therefore an important current goal is to clarify these observations. Functionally, the role of interaction with actin is becoming clearer from a combination of in vitro and in vivo studies. In vitro motility assays, biochemical ATPase assays and single molecule studies have revealed a cMyBP-C induced sensitization of thin filament activation to calcium consistent with a left shift of the velocity-pCa curve (Belknap et al. 2014; Mun et al. 2014; Previs et al. 2012; Razumova et al. 2006; Saber et al. 2008). However, at high calcium cMyBP-C slowed down actin or thin filament sliding and reduced myosin’s ATPase activity. Note, however, that these in vitro assays often use high concentrations of cMyBP-C resulting in high levels of actin saturation. Altogether this suggests a model where cMyBP-C binding at low [Ca^2+^] displaces tropomyosin towards the “closed” position, while at high Ca^2+^ cMyBP-C blocks or competes with S1 binding, or creates a viscous load to reduce sliding (Craig et al. 2014; Mun et al. 2014; Walcott et al. 2015). The role of cMyBP-C competing with myosin binding will be different in the sarcomere where the stoichiometry of myosin:cMyBP-C:actin will limit competition between myosin and MyBP-C. Interestingly, in mouse the effects of tropomyosin displacement were highly cMyBP-C domain specific. Only C0C3 caused displacement and increased sensitivity to Ca^2+^ while shorter N-terminal fragments (C0C1 and C0C1f containing the first 17M-domain residues) did show thin filament binding but no effect on the Ca^2+^ sensitivity and S1 binding (Belknap et al. 2014; Inchingolo et al. 2019; Mun et al. 2014). This highlights that a clear definition of both long-lived and transient binding sites on actin for cMyBP-C are required.

## The governing role of cMyBP-C

The role of cMyBP-C is fascinating in its complexity, but its function can potentially be distilled into two actions, governing activation through thin filament interactions, and governing force by controlling myosin head availability through the thick filament. As described above, cMyBP-C is capable of sensitizing the thin filament to myosin binding at low calcium; this has been visualized as a shift in tropomyosin towards the more active ‘closed’ state (Mun et al. 2011, 2014). However, in these studies, despite visualizing the tropomyosin shift, cMyBP-C was not observed as a clear density instead only the proximal region was visible. Recently, cryo-EM studies were able to visualize the whole of the N-terminal fragment bound to the thin filament (Risi et al. 2018). This difference may be due to the lower levels of decoration used in the negative stain experiments resulting in loss of apparent density due to averaging, or could reflect a dynamic interaction between cMyBP-C and the thin filament; the latter has recently been directly observed (Inchingolo et al. 2019). Imaging fluorescent C0C3 showed a dynamic search along the thin filament, potentially offering a mechanism for sensing its activation state. At high calcium fewer molecules were observed to diffuse, instead binding more strongly, competing with myosin binding and also perhaps providing a viscous load to slow velocity (Inchingolo et al. 2019). These two distinct states, one dynamic and the second static may explain the two binding modes observed using electron microscopy. Indeed, Risi et al. (2018) suggest that C1 presents a strong interaction with actin further enhanced by C0, potentially offering a direct structural assignment of these two activities.

Activation of the thin filament provides the trigger to enable contraction, however the force exerted by the myosins is dependent on the number of available heads. This second aspect of cMyBP-C’s function is complex and likely involves distinct mechanisms to release heads e.g. through phosphorylation or force-induced. The precise origin of the latter, force-induced effects are hotly debated. Driven by x-ray diffraction and fluorescence polarization studies, it remains unclear whether cMyBP-C’s function as a force transducer between thick and thin filaments, or simply a modulator of myosin head availability (Caremani et al. 2019b; Fusi et al. 2017; Irving and Craig 2019; Kampourakis et al. 2014; Linari et al. 2015; Reconditi et al. 2017). These findings are further confounded by the recent observation that heads released from the thick filament may not necessarily be in an active state (Caremani et al. 2019a).

Clearly cMyBP-C is an important modulator of cardiac output, at one level working to release heads to increase the force generating capacity of the heart, but then to sequester these heads in a low energy usage state when not required. However, the extent of cooperative force-dependent head release for interaction with the thin filament, and cMyBP-C’s role in this, is unknown. The differential binding of cMyBP-C N-terminal regions to both myosin and to actin suggests a very important regulatory role for this protein, shuttling between thick and thin filaments, modulated by phosphorylation/load. In a very recent study, this was investigated at multiple levels to demonstrate that hierarchical phosphorylation imbues specific properties on cMyBP-C. At low phosphorylation levels the myosin heads are released and cMyBP-C can participate in thin filament activation, whereas at higher phosphorylation levels the activation of actin is blunted, facilitating diastole (Ponnam et al. 2019). This has added a more nuanced appreciation of earlier data showing high levels of phosphorylation decreased the interaction between cMyBP-C and the thin filament (Previs et al. 2016; Shaffer et al. 2009; Weith et al. 2012a, b). However, at high calcium levels the structure of phosphorylated cMyBP-C reverts to one capable of binding the thin filament by direct calcium binding to cMyBP-C (Previs et al. 2016).

These complex interactions make it difficult to tease apart the distinct roles of cMyBP-C, and to understand their importance with the context of the stoichiometric and spatial constraints of the sarcomere. The latter are further exacerbated by the inconsistent alignment between thick and thin filament periodicities (43 and 36 nm, respectively) resulting in clear landing zones for these proteins, as demonstrated for myosin and actin using laser tweezers (Steffen et al. 2001). To more adequately extract an understanding of this complex system will require an appreciation of the activation timescales induced by force, RLC phosphorylation and cMyBP-C phosphorylation. Disentangling this time response may well clarify the roles of thick and thin filament responses to stimulation. In addition, these timescales need to be woven into the thin filament state switching which occurs rapidly, consistent with a similar affinity of TnI for TnC (McKay et al. 1997) in response to Ca^2+^ and myosin binding (McKillop and Geeves 1993). Phosphorylation of MyBP-C (and other thick and thin filament components) is likely a slower response resulting in a gross change in the contractile capacity of the sarcomere, calcium sensitivity and rates of activation and relaxation. Altogether, these observations highlight the complex role of cMyBP-C in the sarcomere and explain why this protein is such a prolific target of pathogenic mutation.

## Does MyBP-C co-orchestrate actomyosin interactions?

**Fig. 4:**
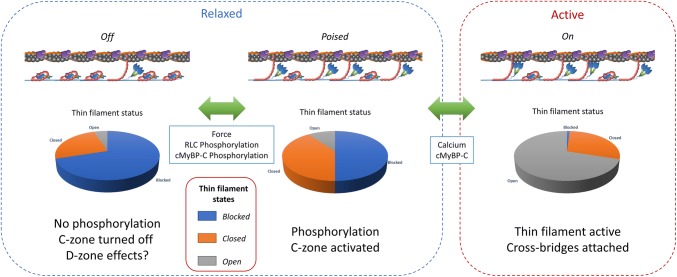
The interplay between the thick and thin filaments mediated by cMyBP-C. The relaxed state is represented as a continuum of thick filament and thin filament states, which are modulated by phosphorylation and/or force. In a completely relaxed condition very few C-zone heads are likely to be activated, at the other end of the spectrum with RLC, cMyBP-C phosphorylation, and force the number of heads ready for generating tension is maximized. Interestingly, even though phosphorylation of cMyBP-C releases heads from the thick filament, it prevents cMyBP-C interacting with the thin filament. The acute stage of activation, mediated by calcium binding to the thin filament, results in cross-bridge attachment and now cMyBP-C can reveal its second, modulatory role on contraction. The complexity of this process is grossly underestimated in this diagram, however the questions necessary to understand this process are revealed

The more we seem to understand about cMyBP-C the more challenging a model is required to describe its function. We have summarised many of the observations in Fig. 4, showing how the relaxed state is a continuum of states capable of modulating the force once the thin filament is activated. The highlighted mechanisms of activation show how the interaction between the thick and thin filaments are inter-dependent and how cMyBP-C lies at the core of this. Since cMyBP-C has the ability to bind both myosin and actin with similar affinities, the mechanisms employed in vivo to regulate its binding partners are crucial to understand (Wang et al. 2016). The stoichiometry and cooperativity of the contacts between cMyBP-C and myosin/actin need to be determined across a range of conditions. This will reveal if release of heads from the thick filament is cooperative and also how titin plays a role in this (presumably as a force conductor). A long-established difference exists between the cooperativity of velocity (e.g. in vitro motility) and tension. The latter has a Hill coefficient typically above 5 and motility is usually below 2. Perhaps the activating effects of cMyBP-C on the thin and thick filaments can reconcile this difference, but how is this regulated? Is there a direct effect of calcium on the thick filament or is this via a different mechanism?

Despite these potential mechanisms of activation, other fundamental questions regarding the localisation of cMyBP-C in the C-zone also need to be addressed. The diversity of cMyBP-C binding partners might suggest a role in sarcomeric organisation and repair, or to offset the calcium gradient in the cardiac sarcomere (Previs et al. 2015). The latter is of interest when considering other muscle types where the T-tubules are located away from the Z-line.

Finally, the molecular picture provided in this review provides a framework that will ultimately require explanation at the physiological contraction level. Such observations show inconsistencies between studies possibly due to the method of study or sample preparation which may affect lattice spacing (Irving and Craig 2019), or inhomogeneities due to phosphorylation between myocyte preparations. To achieve such connectivity on multiple scales will require uniform sample preparation from one source and computational models that scale between such levels (Chase et al. 2004; Mijailovich et al. 2016; Niederer et al. 2019; Tanner et al. 2008; Walcott et al. 2015; Wang et al. 2016).

The continuing story of cMyBP-C is providing a revelation in our understanding of muscle, both in normal and disease conditions. With the advancements in imaging and spectroscopy technologies across a range of resolutions both in vivo and in vitro it is hoped that these can be provide a detailed time-resolved view of cMyBP-C function. Reconciling a stronger molecular understanding with physiological observations using computational methods offers a mechanism to solve the remaining questions of how cMyBP-C modulates contraction and why it is such a prolific site of mutation in disease.
